# Erythrocytosis is associated with intradialytic hypotension: a case series

**DOI:** 10.1186/s12882-019-1426-7

**Published:** 2019-07-02

**Authors:** Shree Agrawal, Preethi Ramachandran, Rupinder Gill, Samuel Spitalewitz, Douglas Gunzler, Marcia R. Silver, Edward J. Horwitz, Jeffrey R. Schelling

**Affiliations:** 1 Case Western Reserve University School of Medicine, 2109 Adelbert Road, Cleveland, OH 44016 USA; 2 Department of Medicine, MetroHealth Medical Center, 2500 MetroHealth Drive, Cleveland, OH 44109 USA; 3 Brookdale University Hospital Medical Center, Brooklyn, NY 11212 USA; 4 MetroHealth Medical Center Center for Health Care Research and Policy, Case Western Reserve University, 2500 MetroHealth Drive, Cleveland, OH 44109 USA

**Keywords:** End stage renal disease, EPO, Erythrocytosis, Renal cysts, thrombosis

## Abstract

**Background:**

For patients with end stage renal disease undergoing hemodialysis, erythrocytosis occurs rarely. Erythrocytosis increases the risk of thrombosis, which is a common complication in hemodialysis patients. The risk of thrombosis may also be increased by hypotension. The purpose of our report is to examine the relationship between intradialytic hypotension and erythrocytosis.

**Case presentation:**

We present a series of five patients with end stage renal disease and erythrocytosis (peak hemoglobin range 15.2–18.5 g/dL). All were erythropoiesis-stimulating agent naïve and non-smokers. Prior to developing erythrocytosis, each patient developed recurring episodes of intradialytic hypotension over several months. A statistically significant inverse correlation was observed between nadir intradialytic systolic blood pressure and hemoglobin concentration. In the index case, midodrine treatment resulted in resolution of the hypotension and erythrocytosis. Most of the patients had multiple acquired renal cysts, which is a potential source of erythropoietin. Four of the five cases developed arteriovenous dialysis access or deep venous thrombosis.

**Conclusions:**

An association between intradialytic hypotension and erythrocytosis was observed in five cases. We postulate that chronic intermittent hypotension and renal ischemia may lead to erythropoietin secretion, and this cascade could represent a newly recognized cause of secondary erythrocytosis.

## Background

Erythrocytosis is rare in the context of end stage renal disease (ESRD). The most common erythropoietin (EPO)-dependent etiologies of erythrocytosis include malignancies, chronic hypoxia and obstructive sleep apnea, while myeloproliferative disorders account for most cases of EPO-independent erythrocytosis. However, in many instances, the mechanism of erythrocytosis in ESRD patients is unknown. Renal EPO production is stimulated by decreased oxygen carrying capacity (hemoglobin concentration x O_2_ saturation x cardiac output) [1–6]. Because the ability to secrete EPO is diminished in chronic kidney diseases, most ESRD patients require erythropoiesis-stimulating agents (ESA) to prevent anemia [7]. We describe five ESA-independent patients with ESRD and erythrocytosis, and associated chronic intermittent hypotension, in accordance with established case report guidelines [8].

## Case presentation

The index case was a 57-year old man with ESRD due to diabetic kidney disease, on hemodialysis for 14 years. Other medical problems included hypertension for > 30 years, peripheral neuropathy and multiple arteriovenous dialysis access revisions. Medications were valsartan, amlodipine, doxazosin, metoprolol, cinacalcet, lanthanum carbonate, pantoprazole, zolpidem, and vitamin D2. Blood pressure was managed with three to four medications for many years. There was a remote history of sleep apnea that resolved after 45 kg weight loss, and no history of smoking or COPD.

Physical examination (after erythrocytosis developed) revealed blood pressure 126/74, pulse 84, dry weight 93 kg, body mass index 30.4 kg/m^2^. Head and neck examination, cardiac, respiratory and abdominal exam were normal. Extremities showed 2+ symmetric pulses, no peripheral edema, and non-functioning dialysis grafts in his right and left upper arms and left thigh, with a right femoral tunneled dialysis catheter in place. Neurologic exam revealed diminished sensation to pinprick and altered proprioception in both feet.

**Fig. 1:**
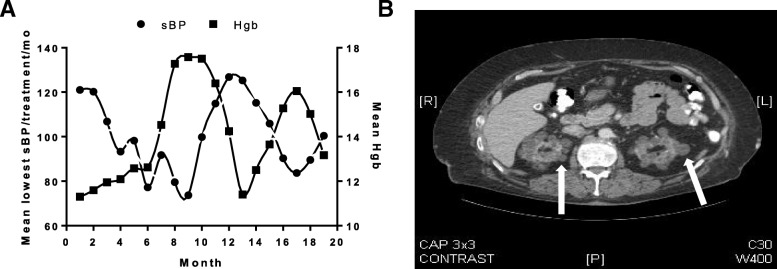
Clinical data about the index case. **a** Temporal relationship between mean nadir systolic blood pressure (measured every 30 min with automated devices associated with the dialysis machine, and averaged monthly from the lowest value during each dialysis treatment) and hemoglobin concentration (mean from two to four values per month). **b** Abdominal computed tomography (CT) scan revealing multiple, bilateral acquired renal cysts (arrows)

The patient experienced recurrent episodes of asymptomatic intradialytic hypotension (Fig. 1a), which persisted despite gradually increasing his dry weight to 97 kg and discontinuing anti-hypertensive medications between months 3–7. In month 8, midodrine 5 mg by mouth for blood pressure support was started prior to each dialysis session, increasing to a second 5 mg dose after two hours of dialysis in month 9. This resolved the intradialytic hypotension. Midodrine was discontinued in month 14, causing relapsing hypotension; midodrine reinstitution in month 17 resolved the hypotension again (Fig. 1a).

Investigation for the cause of hypotension included an echocardiogram, which demonstrated concentric left ventricular hypertrophy, 55% left ventricular ejection fraction, normal right ventricular function, and absence of pulmonary hypertension, pericardial effusion or valve defects. Adrenal and thyroid function tests were normal. An endocrinology consultant concluded that the hypotension was due to diabetic autonomic neuropathy.

**Fig. 2:**
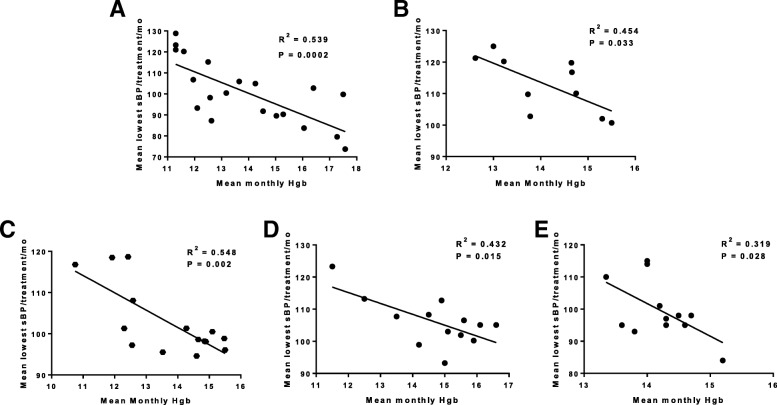
Analyses of blood pressure and hemoglobin concentration in five chronic hemodialysis patients with unexplained erythrocytosis. **a**-**e** Relationship between mean monthly hemoglobin concentrations and mean nadir systolic blood pressure during dialysis. All blood pressures were recorded with automated devices associated with the dialysis machines. Each graph represents data from an individual patient. Linear regression and Pearson R^2^ values were calculated using GraphPrism software

Prior to the intradialytic hypotension episodes, the hemoglobin concentration ranged between 10.3–12.0 g/dL. The patient never required ESA therapy. The patient’s hemoglobin concentration increased over six months, peaking at 18.5 g/dL. The temporal relationship between monthly mean nadir blood pressure on dialysis and hemoglobin concentration is shown in Figs. 1a and 2a.

Between months 6–10, the patient experienced thrombosis of the right brachiocephalic and left femoral grafts. In month 8, he was diagnosed with a deep venous thrombosis involving the left posterior tibial and proximal superficial femoral veins, which was treated with warfarin.

Laboratory investigation demonstrated normal platelet counts, peripheral blood smear, and partial thromboplastin times, EPO level of 100.0 mIU/mL (normal range 3.7–28.4), negative factor V Leiden and negative JAK2 (V167F) gene mutations. Computed tomography of the patient’s chest, abdomen and pelvis revealed no renal or liver masses, but did show multiple, bilateral kidney cysts (Fig. 1b).

### Additional cases

Queries to nephrologists in two large groups revealed four additional subjects with ESRD and unexplained (non-smokers without COPD, obstructive sleep apnea, malignancy, ESA or iron therapies) sustained hemoglobin concentration > 13 g/dL for > 6 months (Table 1). All patients had prolonged episodes of intradialytic hypotension over several months, which preceded the erythrocytosis. Three of the five subjects had documented acquired renal cysts, and multiple vascular access thromboses (Table 1).

**Table 1:** Demographic and clinical information

Patient ID	Age	Gender	ESRD duration (years)	ESRD cause	Renal cysts	Multiple dialysis vascular access thrombosis	Co-morbid conditions
Index	57	M	14	Diabetes	Yes	Yes	Hypertension, DVT, autonomic neuropathy, remote OSA
2	48	M	6	Diabetes	No	Yes	Retinopathy, hypertension, hyperlipidemia, heart failure, remote pulmonary embolism
3	58	M	6	Unclear	Yes	Yes	HIV, hepatitis C, hypertension
4	40	M	2	Unclear	Yes	No	Hypertension
5	45	F	6	Unclear	No	Yes	Hypertension

Abbreviations: *M* male, *F* female, *ESRD* end stage renal disease, *DVT* deep venous thrombosis, *OSA* obstructive sleep apnea, *HIV* human immunodeficiency virus

The relationships between nadir intradialytic systolic BP and mean monthly hemoglobin concentration for all five patients are plotted in Fig. 2. In each case, linear regression revealed a statistically significant (*P* < 0.05) inverse correlation between blood pressure and hemoglobin concentration. To further evaluate the association between hypotension and hemoglobin concentration, a comparative analysis was conducted using average monthly nadir systolic BP as a continuous, independent variable and hemoglobin concentration as the predefined binary outcome (months when mean hemoglobin concentration < 13 g/dL or ≥ 13 g/dL). We employed a multilevel generalized growth model with a logit link approach (MPlus software, 2012) which accounts for within-subject variability in BP and hemoglobin concentrations. Lower BP was associated with a significantly increased odds ratio for a change from low to high hemoglobin concentration (OR = 1.163, 95% CI = 1.090, 1.241).

## Discussion and conclusions

We report five cases of sustained intradialytic hypotension and erythrocytosis in patients with ESRD on hemodialysis. In all cases, hypotension preceded the increased hemoglobin concentration. In the index case, both intradialytic hypotension and erythrocytosis resolved with midodrine, recurred with midodrine discontinuation, and improved with midodrine reinstitution. Statistically significant inverse correlations between nadir intradialytic blood pressure and hemoglobin concentration were observed using two different statistical methods.

The index case had diabetic autonomic neuropathy as a major contributing factor to his dialysis-associated hypotension. Primary autonomic neuropathy and type 2 diabetes have each been associated with EPO deficiency and anemia [9, 10]. Results from studies of diabetic autonomic neuropathy are conflicting, and the interpretation can be confounded by concomitant diabetic nephropathy, which may contribute to diminished renal EPO production. In a report of patients with diabetic autonomic neuropathy and preserved glomerular filtration rate, hemoglobin and autonomic neuropathy score were modestly, but inversely related [11]. In a canine model of simulated autonomic neuropathy with renal sympathetic denervation, a hypoxic stimulus with acute hemorrhagic shock resulted in accentuated decreases in renal blood flow, and subsequent stimulation of EPO secretion [12]. These data suggest that hypotension and ischemia precipitate erythropoiesis.

Symptomatic hypotensive episodes during hemodialysis occur in approximately 9% of patients, primarily due to ultrafiltration and rapid reductions in circulating blood volume [13, 14], although autonomic dysfunction, myocardial infarction and drugs represent additional common causes. Renal blood flow autoregulation is impaired in animal models of diabetic and hypertensive chronic kidney disease, indicating that overt hypotension, as well as blood pressures in the low normal range are likely associated with inadequate renal perfusion during hemodialysis [15]. Since EPO is physiologically regulated by O_2_-carrying capacity (cardiac output x O_2_ saturation x hemoglobin concentration), we speculate that frequent hypotensive episodes, due to decreases in cardiac output while on dialysis, could represent a stimulus for EPO secretion. We cannot be certain that hypoxia stimulates biochemical pathways in EPO-producing cells in end stage kidneys, but there is a report of sleep apnea associated with erythrocytosis in an ESRD patient, which resolved with CPAP [4], implying that it is possible. Because none of the ESRD patients in our series had ever received ESA therapy, this suggests some residual, endogenous EPO secretion remained intact, which could represent part of the profile for susceptibility to hypoxia-induced erythrocytosis.

Midodrine is an α1-adrenergic agonist, which causes vasoconstriction of arteriolar and venous capacitance vascular beds, leading to enhanced venous return and cardiac output [16]. Midodrine is an effective and safe treatment for intradialytic hypotension [17–19], with no evidence that midodrine directly causes anemia or changes in hemoglobin concentration [19]. Although detailed documentation of midodrine treatment was only available for our index patient, there was a striking temporal relationship between initial midodrine treatment and re-challenge, with suppression of hemoglobin concentration during midodrine treatment.

Four of the five patients with recurrent hypotension and erythrocytosis developed a deep venous thrombosis and/or dialysis vascular access thrombosis. In studies of non-dialysis patients with orthostatic hypotension and syncope, von Willebrand factor activity was significantly elevated, thereby supporting a procoagulant state [20], and patients with idiopathic erythrocytosis or polycythemia vera are also at increased risk for hyperviscosity and thrombotic events [21, 22]. EPO also has been shown to cause vasoconstriction, thrombosis and endothelial damage, suggesting that intermittent, sustained hypotension and diminished renal blood flow during dialysis could stimulate compensatory EPO-induced vasoconstriction, or serve as the primary cause of renal blood flow reduction, independent of hemoglobin or O_2_ saturation cues [23, 24].

Three of the five patients in our series had multiple bilateral renal cysts, which have been associated with increased EPO among hemodialysis patients [3]. One study reported that erythrocytosis completely resolved after therapeutic puncture of a giant renal cyst [5], and suggested that excess EPO was produced by interstitial cells within the stroma of the cyst wall. In our series, we speculate that the EPO-producing cells are not autonomous, but remain sensitive to hypoxic cues, resulting in transcriptional EPO upregulation. Accurate verification of the EPO source would require cyst fluid and/or renal vein sampling, which we considered an unjustifiable risk, particularly in the patients requiring warfarin therapy. However, we excluded hepatic masses in all cases, which are the most common source of extrarenal EPO. Finally, because some patients in our series did not have acquired renal cysts, this cannot be the sole cause of erythrocytosis in all cases.

The limitations of our case series include the observational and retrospective study design, which render causation difficult to establish. Although there was a temporal and statistically significant correlation between blood pressure and hemoglobin concentration, the sample size was small, and we cannot exclude that factors other than hypotension and ischemia (e.g., undiagnosed sleep apnea) also contributed to erythrocytosis. In addition, patients were specifically selected because of their history of unexplained elevated hemoglobin concentrations, and these conclusions therefore may not be applicable to all patients undergoing dialysis. Finally, most dialysis patients with acquired renal cysts and/or hypotension do not develop erythrocytosis. However, intradialytic hypotension is generally transient, and resolves with adjustments of the dialysis prescription. Our cohort is unique, in that prolonged durations of hypotension episodes occurred frequently and over many months.

We are unaware of prior descriptions of associated hypotension and erythrocytosis in chronic hemodialysis patients. Since the patients in this series developed hypotension that preceded and correlated with erythrocytosis, and the index patient’s episodes resolved with anti-hypertensive medication discontinuation and midodrine therapy as the sole interventions, we speculate that intermittent decreased renal perfusion and hypoxia could have been the triggers for erythropoiesis. The resulting combination of hypotension and erythrocytosis formed a strong stimulus for thrombotic events. We therefore propose that vigilance for this syndrome and treatment with vasoconstrictive agents may prevent life-threatening thromboembolic complications in vulnerable dialysis patients.

## Data Availability

De-identified source data and material would be provided by the corresponding author upon request.

## Abbreviations

CT: Computed tomography
EPO: Erythropoietin
ESA: Erythropoiesis-stimulating agent
ESRD: End stage renal disease
